# Maternal L-Carnitine Supplementation Improves Brain Health in Offspring from Cigarette Smoke Exposed Mothers

**DOI:** 10.3389/fnmol.2017.00033

**Published:** 2017-02-13

**Authors:** Yik Lung Chan, Sonia Saad, Ibrahim Al-Odat, Brian G. Oliver, Carol Pollock, Nicole M. Jones, Hui Chen

**Affiliations:** ^1^Center for Health Technologies, School of Life Sciences, Faculty of Science, University of Technology SydneyUltimo, NSW, Australia; ^2^Respiratory Cellular and Molecular Biology, Woolcock Institute of Medical Research, The University of SydneyGlebe, NSW, Australia; ^3^Renal Group Kolling Institute, Royal North Shore HospitalSt Leonards, NSW, Australia; ^4^Department of Pharmacology, School of Medical Sciences, University of New South WalesKensington, NSW, Australia

**Keywords:** maternal smoking, autophagy, mitophagy, oxidative stress, gender difference

## Abstract

Maternal cigarette smoke exposure (SE) causes detrimental changes associated with the development of chronic neurological diseases in the offspring as a result of oxidative mitochondrial damage. Maternal L-Carnitine administration has been shown to reduce renal oxidative stress in SE offspring, but its effect in the brain is unknown. Here, we investigated the effects of maternal L-Carnitine supplementation on brain markers of oxidative stress, autophagy, mitophagy and mitochondrial energy producing oxidative phosphorylation (OXPHOS) complexes in SE offspring. Female Balb/c mice (8 weeks) were exposed to cigarette smoke prior to mating, during gestation and lactation with or without L-Carnitine supplementation (1.5 mM in drinking water). In 1 day old male SE offspring, brain mitochondrial damage was suggested by increased mitochondrial fusion and reduced autophagosome markers; whereas at 13 weeks, enhanced brain cell damage was suggested by reduced fission and autophagosome markers, as well as increased apoptosis and DNA fragmentation markers, which were partially reversed by maternal L-Carnitine supplementation. In female SE offspring, enhanced mitochondrial regeneration was suggested by decreased fission and increased fusion markers at day 1. At 13 weeks, there was an increase in brain energy demand, oxidative stress and mitochondrial turnover, reflected by the protein changes of OXPHOS complex, fission and autophagosome markers, as well as the endogenous antioxidant, which were also partially normalized by maternal L-Carnitine supplementation. However, markers of apoptosis and DNA fragmentation were not significantly changed. Thus L-Carnitine supplementation may benefit the brain health of the offspring from smoking mothers.

## Introduction

Cigarette smoking is a leading cause of death and morbidity worldwide. Despite increased public education and government policies to ban smoking in public places (Balmford et al., 2016), smoking among women of childbearing age and during pregnancy is still common (Mendelsohn et al., 2014). This is partially due to low success rates of smoking cessation during pregnancy (Glover et al., 2016). The adverse impact of maternal smoking on health outcomes in the next generation has been well studied, including increased risk of type 2 diabetes mellitus, impaired renal function and structure, and sudden infant death (Jaakkola and Gissler, 2004; Shah et al., 2006; Al-Odat et al., 2014; Fang et al., 2015). In addition, we have shown increased inflammation, abnormal mitochondrial metabolic markers and oxidative stress-related cell injury in the brains of offspring from cigarette smoke exposed (SE) mothers (Chen et al., 2011; Chan et al., 2016b).

Autophagy removes damaged or junk organelles in cells (Ashrafi and Schwarz, 2013). Mitophagy is when autophagy occurs in the mitochondria, which is an important quality control mechanism to remove damaged mitochondria (Ashrafi and Schwarz, 2013). This process recycles intact mitochondrial fragments to generate new healthy mitochondria through fission and fusion (Bereiter-Hahn, 1990; Westermann, 2010) to maintain mitochondrial integrity. In stroke, reduced mitophagy and autophagy may hinder the prompt clearance of damaged mitochondria in the brain, leading to a loss of protection from increased reactive oxygen species (ROS) and reduced mitochondrial ATP supply (Frugier et al., 2016). A reduction in autophagy to clear aggregated proteins appears to underlie the development of neurodegeneration in Parkinson’s disease (Zhang et al., 2015). Our previous data also suggests that maternal SE impairs brain mitochondrial levels of oxidative phosphorylation (OXPHOS) complexes in male offspring with reduced endogenous antioxidant capacity from birth (Chan et al., 2016b). This is due to the changes of mitochondrial dynamics in the brain from birth to adulthood (Hagberg et al., 2014). However, the changes in markers of autophagy and mitophagy in this process are unclear. This formed the first aim of this study.

Furthermore, as SE offspring have reduced brain antioxidants and increased cellular oxidative damage (Chan et al., 2016b), boosting the antioxidant capacity during early life may ameliorate the impact of maternal SE on brain outcomes. The antioxidant L-Carnitine has been shown to improve white matter lesion after chronic hypoperfusion in rats (Ueno et al., 2015) and can provide neuroprotection by elevating brain antioxidant capacity in aging rats (Juliet et al., 2005). We have shown that maternal L-Carnitine supplementation during pregnancy and lactation can alleviate oxidative stress, as well as mitochondrial and renal dysfunction in offspring from SE mothers (Nguyen et al., 2015). As such, this approach may also ameliorate the impact of maternal SE on the brain by affecting mitophagy and autophagy markers. In this study, we investigated the impact of maternal L-Carnitine supplementation during gestation and lactation on brain markers of mitophagy, autophagy, mitochondrial antioxidant and OXPHOS complexes I–V in SE offspring of both genders.

## Materials and Methods

### Animals

The animal experiments were approved by the Animal Care and Ethics Committee at the University of Technology Sydney (ACEC#2011-313A). All protocols were performed according to the Australian National Health and Medical Research Council Guide for the Care and Use of Laboratory Animals. Female Balb/c mice (8 weeks, Animal Resources Centre, Perth, WA, Australia) were housed at 20 ± 2°C and maintained on a 12 h light, 12 h dark cycle (lights on at 06:00 h) with *ad libitum* access to standard rodent chow and water. After the acclimatization period, mice were assigned to sham exposure (SHAM), and SE groups. The SE group was exposed to two cigarettes (Winfield Red, ≤1.2 mg nicotine; VIC, Australia) in a perspex chamber (15L), twice daily for 6 weeks prior to mating, during gestation and lactation; while the SHAM group was exposed to air during the same period of time as previously described (Al-Odat et al., 2014). For each session, the mice were exposed the smoke from one cigarette for 15 min with a 5-min interval between two cigarettes. Female breeders were mated with males (8 weeks) from the same source, which were not exposed to cigarette smoke. Half of the SE breeders were continuously supplied with L-Carnitine (SE breeders supplied with L-Carnitine [SELC], 1.5 mM directly dissolved in drinking water) during gestation and lactation periods as previously described (Nguyen et al., 2015). L-Carnitine dose was determined according to a previous publication (Ratnakumari et al., 1995). Normal drinking water was provided to the SHAM and SE dams. Brains from offspring of both genders were collected at postnatal (P) day 1 (male = 17; female = 20), P20 (male = 14; female = 10) and 13 weeks (male = 10; female = 8). P1 mice were sacrificed by decapitation, while animals older than 20 days were sacrificed by anesthetic overdose (Pentothal^®^, 0.1 mg/g, i.p., Abbott Australasia Pty. Ltd., Macquarie Park, NSW, Australia) between 9:00–12:00 h. The brains were stored at −80°C for protein analysis.

### Western Blotting

The protein levels of dynamin-related protein (Drp)-1, fission protein (Fis)-1, phosphatase and tensin homolog induced putative kinase (Pink)-1, Parkin, optic atrophy (Opa)-1, light chain 3 microtubule-associated protein A/B (LC3A/B), manganese superoxide dismutase (MnSOD), translocase of outer membrane (Tom)-20 and OXPHOS complexes were measured by western blotting. Brains were homogenized using lysis buffer for whole protein and mitochondrial protein extraction as previously described (Nguyen et al., 2015). Protein samples (20 μg) were separated on NuPage^®^ Novex^®^ 4%–12% Bis-Tris gels (Life Technologies, Carlsbad, CA, USA), then transferred to PVDF membranes (Rockford, IL, USA), which were blocked with non-fat milk and incubated with primary antibodies (OXPHOS complexes; 1:2500, Abcam, Cambridge, UK), Drp-1 (1:2000, Novus Biotechnology, Littleton, CO, USA), Opa-1 (1:2000, Novus Biotechnology, Littleton, CO, USA), LC3A/B (1:2000, Cell Signaling Technology, Danvers, MA, USA), Tom-20 (1:2000, Santa Cruz Biotechnology, Dallas, TX, USA) and MnSOD (1:2000, Millipore, Billerica, MA, USA), Pink-1 (1:1000, BioVision Incorporated, Milpitas, CA, USA), Fis-1 (1:500, Santa Cruz Biotechnology, Dallas, TX, USA) and Parkin (1:500, Cell Signaling Technology, Danvers, MA, USA) for overnight. Membranes were then incubated in secondary antibodies (goat anti-rabbit or rabbit anti-mouse IgG horseradish peroxidase-conjugated secondary antibodies, 1:5000 for OPA-1, MnSOD, Tom-20, OXPHOS complexes; 1:2000 for Drp-1, Pink-1, LC3A/B; 1:500 for Parkin; Santa Cruz Biotechnology, Dallas, TX, USA) for 1 h. Protein expression was detected by SuperSignal West Pico Chemiluminescent substrate (Thermo, Waltham, MA, USA) and Fujifilm LAS-3000 (Fujifilm, Tokyo, Japan). Protein band density was determined using IMAGEJ software (National Institute of Health, Bethesda, MD, USA). Results are expressed as a ratio of the individual marker intensity relative to β-actin or cytochrome c oxidase subunit (Cox) IV band intensity.

### Immunohistochemistry

As this study aimed to investigate the long-term impact of maternal SE and L-Carnitine supplement on the offspring, the brains from the offspring at 13 weeks, representing adulthood, were accessed for apoptosis and DNA damage. Brain sections at bregma—1 mm (*n* = 4 per group) were deparaffinized and treated with xylene and decreasing graded ethanol to distilled water for hydration. The sections were then microwaved for 17 min in citrate buffer (pH 9.0) followed by cooling in water bath for 15 min for heat-induced epitope retrieval. The slides were then quenched with peroxidase (methanol: PBS: H_2_O = 5:5:2) for 15 min at room temperature.

For Caspase-3 staining, the tissues were blocked with 10% normal horse serum for 30 min then incubated with Caspase-3 antibody (1:300, BD Biosciences, Macquarie Park, NSW, Australia) overnight, followed by secondary antibodies (Goat anti-rabbit HRP, 1:200, Vector Laboratories, Burlingame, CA, USA) for 45 min. Diaminobenzidine solution (K346811, DAKO, USA) was then added and incubated for 8 min, followed by counterstaining with Harri’s Hematoxylin, dehydration through graded ethanol to xylene, and coverslipped. Quantification was performed on three slides from each brain blinded to the study groups. Positive cells (brown staining) were counted and the results are represented as the percentage of all cells within a given area.

ApopTag^®^Peroxidase kits (Merck Millipore, Bayswater, VIC, Australia) were used for TUNEL staining. For TUNEL staining, 40 μl of equilibration buffer was added on each section for 30 s after the hydrogen peroxidase quenching step. Terminal deoxynucleotidyl transferase (Tdt, Tdt: reaction buffer = 1:4) was added to each section, coverslipped and incubated for 1 h. Negative control was incubated with water instead of Tdt. Coverslip was then removed and the stop reaction buffer was added. Anti-Digoxigenin-Peroxidase was added to each slide, coverslipped and incubated for 40 min at 37°C. Diaminobenzidine solution (K346811, DAKO, USA) was then added and incubated for 8 min, followed by counterstaining with Harri’s Hematoxylin, dehydration through graded ethanol to xylene, and coverslipped. Quantification was performed on three slides from each brain blinded to study group. Positive cells (brown staining) were counted and the results are represented as the percentage of all cells within a given area.

### Statistical Methods

Results are expressed as mean ± SEM. Normality was tested prior to statistical analysis. If the data were not normally distributed, they were log transformed to research normality. The differences between groups were analyzed using one-way ANOVA followed with Fisher’s LSD test (Statistica 9, Statsoft, Tulsa, OK, USA). *P* < 0.05 was considered significant.

## Results

### Male Offspring

#### Body Parameters

The body weight of the SE offspring was only significantly smaller than SHAM offspring at P1 (*P* < 0.01, Table 1); whereas SELC offspring were heavier than the SE offspring at P1 (*P* < 0.05, Table 1). There were no significant differences in body weights of the male offspring at P20 and 13 weeks among the groups. At P1, SE offspring showed a smaller percentage of brain weight than SHAM offspring (*P* < 0.05, Table 1), which was normalized by maternal L-Carnitine treatment (*P* < 0.01, Table 1). There was no difference in brain weight among the groups at P20 and 13 week (Table 1).

**Table 1:** **Parameters of the male offspring at different ages**.

	Day 1			Day 20			13 weeks		
Offspring	SHAM *n* = 15	SE *n* = 18	SELC *n* = 17	SHAM *n* = 14	SE *n* = 8	SELC *n* = 9	SHAM *n* = 10	SE *n* = 8	SELC *n* = 8
Body weight (g)	1.51 ± 0.06	1.30 ± 0.04**	1.61 ± 0.05^†^	9.97 ± 0.16	9.61 ± 0.14	9.74 ± 0.43	25.6 ± 0.3	24.8 ± 0.3	25.7 ± 0.4
Brain (mg)	10.5 ± 0.3	10.9 ± 0.2	11.1 ± 0.3	26.0 ± 0.3	25.5 ± 0.4	26.2 ± 0.4	29.4 ± 0.4	29.0 ± 0.3	29.5 ± 0.2
Brain%	6.52 ± 0.26	5.84 ± 0.14*	6.92 ± 0.15^††^	2.61 ± 0.04	2.65 ± 0.04	2.73 ± 0.11	1.15 ± 0.02	1.17 ± 0.02	1.15 ± 0.02

Results are expressed as mean ± SEM. Data were analyzed by one way ANOVA. *P < 0.05; **P < 0.01, compared with the SHAM offspring at the same age. ^†^*P* < 0.05; ^††^*P* < 0.01, compared with the smoke exposed (SE) offspring at the same age.

#### Mitophagy Markers

**Figure 1:**
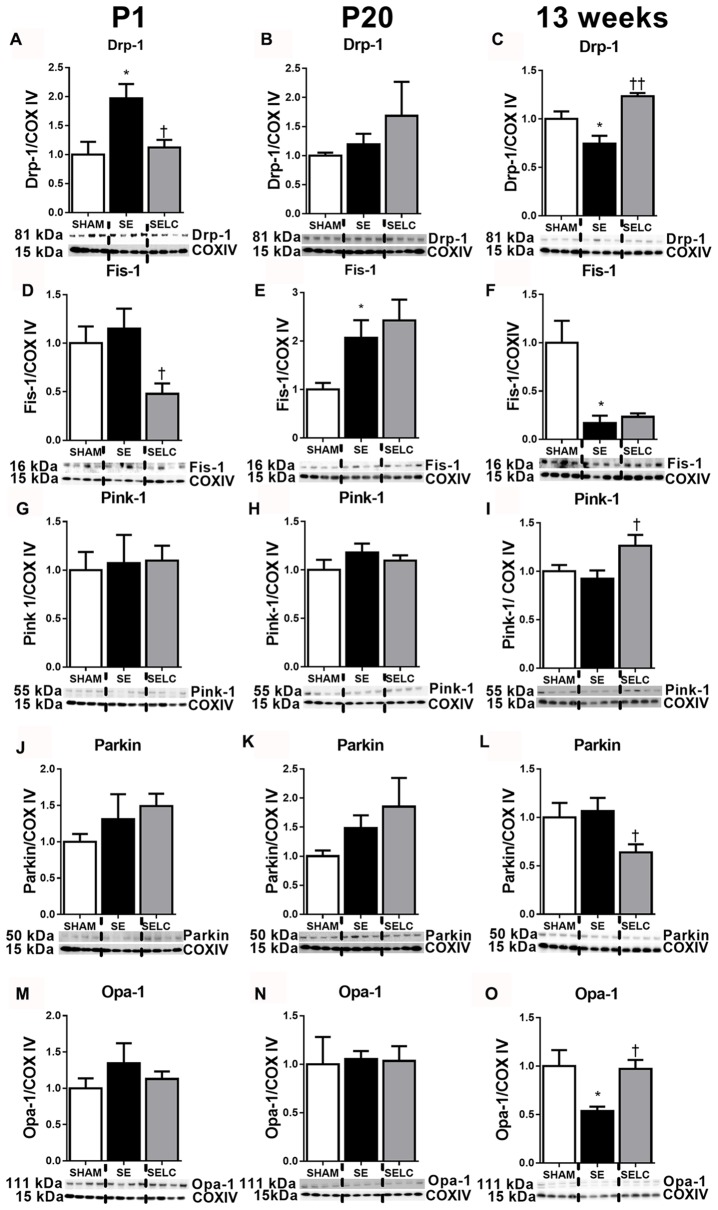
**Brain mitochondria protein levels of Drp-1 (A–C)**, Fis-1 **(D–F)**, Pink-1 **(G–I)**, Parkin **(J–L)** and Opa-1 **(M–O)** in the male SHAM, SE and SELC dams (*n* = 4) at different ages. Results are expressed as mean ± SEM. Data was analyzed by one-way ANOVA with Fisher’s LSD test. *P < 0.05, compared to SHAM; ^†^*P* < 0.05, ^††^*P* < 0.01, compared to SE. Drp-1, dynamin-related protein-1; Fis-1, fission protein-1; Pink-1, phosphatase and tensin homolog (PTEN)-induced putative kinase-1; Opa-1, Optic atrophy-1; Cox IV, cytochrome c oxidase; SE, smoke exposed; SELC, SE with L-Carnitine.

At P1, mitochondrial Drp-1 was increased by 50% in SE offspring compared to SHAM (*P* < 0.05, Figure 1A); while Fis-1, Pink-1, and Parkin and Opa-1 levels were similar between the SHAM and SE offspring (Figures 1D,G,J,M). Maternal L-Carnitine supplementation significantly normalized mitochondrial Drp-1 and reduced Fis-1 levels in the SE offspring (*P* < 0.05, Figures 1A,D), but had no impact on Pink-1, Parkin and Opa-1 proteins at P1 (Figures 1G,J,M). At P20, Fis-1 protein level was increased in the SE offspring, but was not affected by maternal L-Carnitine supplementation (*P* < 0.05, Figure 1E). The other mitochondrial fission and fusion markers were not different among the three experimental groups (Figures 1B,H,K,N). At 13 weeks, brain mitochondrial levels of Drp-1, Fis-1, and Opa-1 were decreased in the SE offspring (*P* < 0.05, Figures 1C,F,O). With L-Carnitine supplementation, mitochondrial Drp-1 and Pink-1 protein level was significantly increased in SELC compared with the SE offspring (*P* < 0.01, Figure 1C); Opa-1 levels were normalized (*P* < 0.05, Figures 1C,I,O). However, Parkin was reduced in the SELC offspring (*P* < 0.01, Figure 1L).

#### Autophagy Markers

**Figure 2:**
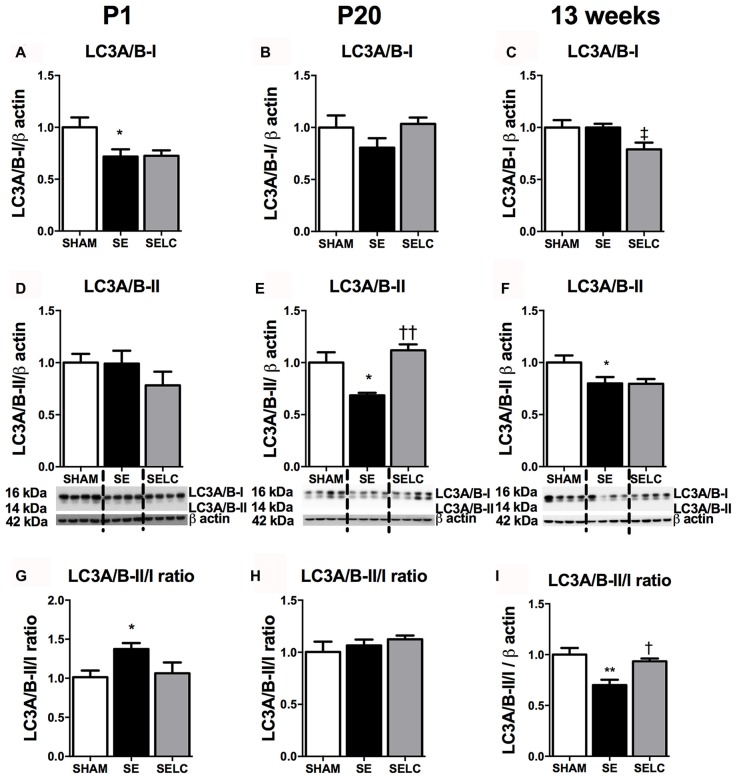
**Brain protein levels of LC3A/B-I (A–C)**, LC3A/B-II **(D–F)**, LC3A/B-II/LC3A/B-I ratio **(G–I)** in the male SHAM, SE and SELC dams (*n* = 4) at different ages. Results are expressed as mean ± SEM. Data was analyzed by one-way ANOVA with Fisher’s LSD test. ^†^*P* < 0.05, ^††^*P* < 0.01, compared to SE. ^‡^*P* < 0.05, compared to SHAM; *P < 0.05, **P < 0.01 compared to SHAM. LC3A/B, Light chain 3 microtubule-associated protein A/B; SE, smoke exposed; SELC, SE with L-Carnitine.

**Figure 3:**
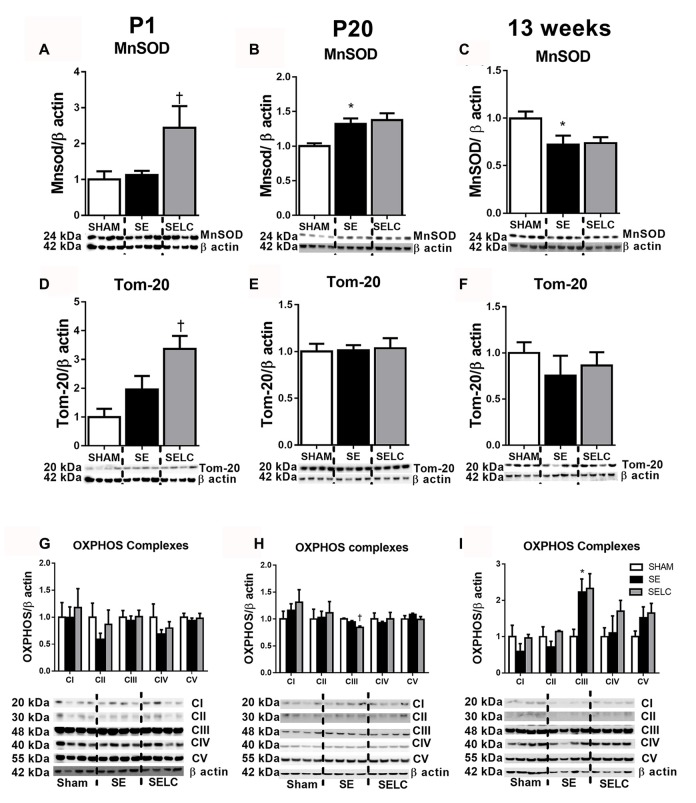
**Brain mitochondria protein levels of MnSOD (A–C)**, Tom-20 **(D–F)**, OXPHOS Complexes I-IV **(G–I)** in the male SHAM, SE and SELC dams (*n* = 4) at different ages. Results are expressed as mean ± SEM. Data was analyzed by one-way ANOVA with Fisher’s LSD test. *P < 0.05, compared to SHAM; ^†^*P* < 0.05, compared SE. MnSOD, manganese superoxide dismutase; Tom-20, translocase of the mitochondrial outer membrane-20; OXPHOS, oxidative phosphorylation; SE, smoke exposed; SELC, SE with L-Carnitine.

At P1, LC3A/B-I was significantly reduced in the SE offspring (*P* < 0.05, Figure 2A). The LC3A/B-II to LC3A/B-I ratio was increased in the SE offspring (*P* < 0.05, Figure 2G), which was normalized in SELC offspring (Figure 2G). At P20, the LC3A/B-II level was significantly lower in SE offspring (*P* < 0.05, Figure 2E), which was normalized by maternal L-Carnitine supplementation (*P* < 0.05 vs. SE offspring, Figure 3E). At 13 weeks, LC3A/B-II level and LC3A/B-II to LC3A/B-I ratio were decreased in SE offspring (LC3A/B-II, *P* < 0.05, Figure 2F; LC3A/B-II/I ratio, *P* < 0.01, Figure 2I), while LC3A/B-II/I ratio was significantly increased in SELC, compared to the SE offspring (*P* < 0.05, Figure 2I). Only LC3A/B-I level was decreased by maternal L-Carnitine treatment (*P* < 0.05, Figure 2C).

#### Mitochondrial Functional Markers

At P1, the mitochondrial Tom-20 level was nearly doubled in SE offspring, although without statistical significance (Figure 3D). MnSOD and mitochondrial OXPHOS complexes were not significantly altered by maternal SE (Figures 3A,D,G). In contrast, L-Carnitine doubled and tripled mitochondrial MnSOD and Tom-20 levels, respectively in the SELC offspring (*P* < 0.05, Figures 3A,D), without having a significant impact on OXPHOS complexes (Figure 3G). At P20, the mitochondrial MnSOD level was increased in the SE compared with SHAM offspring (Figure 3B), while maternal L-Carnitine supplementation only reduced OXPHOS complex III levels (*P* < 0.05, Figure 3H) without affecting the other complex subunits. At 13 weeks, mitochondrial MnSOD was decreased (*P* < 0.05, Figure 3C), while OXPHOS Complex III was significantly increased in the SE offspring (*P* < 0.05 vs. SHAM offspring, Figure 3I). Maternal L-Carnitine had no significant impact on MnSOD, TOM20 and OXPHOS complexes.

#### Cell Apoptosis and DNA Fragmentation

**Figure 4:**
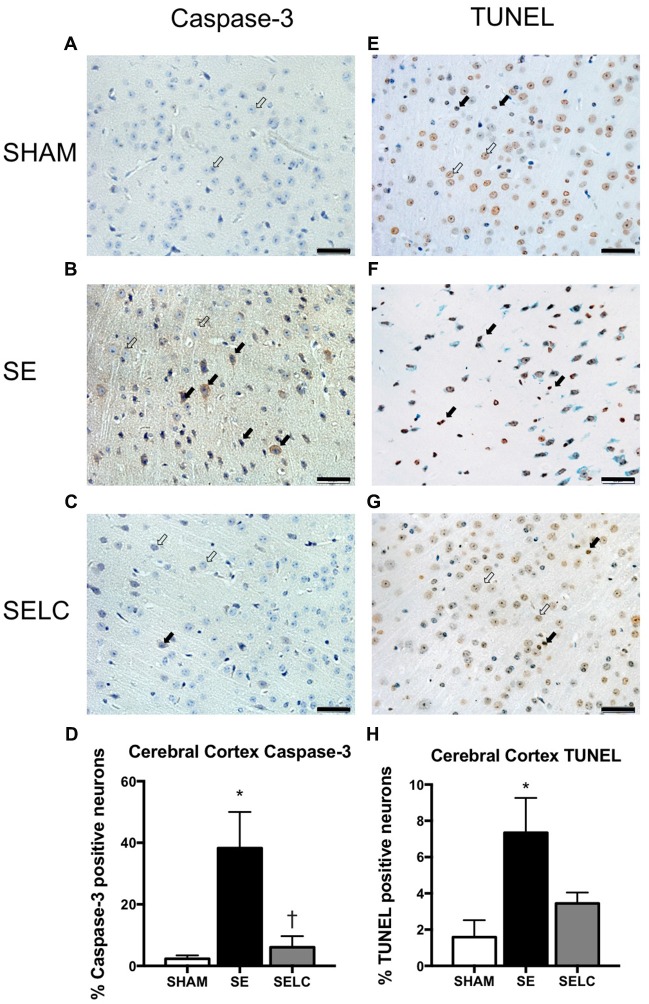
**Immunostaining for Caspase-3 staining in cerebral cortex in the male offspring at 13 weeks (*n* = 4, A–D).** Caspase-3 positive (close arrow) and caspase-3 negative (open arrow). TUNEL staining in cerebral cortex in the male offspring at 13 weeks (*n* = 4, **E–H**). TUNEL positive (close arrow) and TUNEL negative (open arrow). Scale bar = 20 μm. 40× magnification. Results are expressed as mean ± SEM. Data was analyzed by one-way ANOVA with Fisher’s LSD test. *P < 0.05, compared SHAM; ^†^*P* < 0.05, compared to SE. SE, smoke exposed; SELC, SE with L-Carnitine.

At 13 weeks, there was significant increase in caspase-3 and TUNEL positive cell numbers in the cortex of male SE offspring compared with the SHAM offspring (*P* < 0.05, Figures 4D,H). Maternal L-Carnitine treatment normalized caspase-3 level (*P* < 0.05, Figure 4D). Maternal L-Carnitine treatment nearly normalized TUNEL levels although without statistical significance (Figure 4H).

### Female Offspring

#### Body Parameters

The body weight of the SE females was significantly smaller than SHAM offspring at P1, P20 (*P* < 0.05 vs. SHAM, Table 2) and 13 weeks (*P* < 0.01, Table 2). L-Carnitine treatment increased the body weight in SELC offspring at P1 (*P* < 0.01, Table 2), but not at P20 and 13 weeks. Net brain weight was not different between the SHAM and SE offspring, while it was only increased in SELC offspring at P1 (*P* < 0.05 vs. SE offspring, Table 2). The percentage of brain weight was similar among the three groups at P1, P20 and 13 weeks.

**Table 2:** **Parameters of the female offspring at different ages**.

	Day 1			Day 20			13 weeks		
Offspring	SHAM *n* = 11	SE *n* = 20	SELC *n* = 18	SHAM *n* = 8	SE *n* = 11	SELC *n* = 10	SHAM *n* = 8	SE *n* = 8	SELC *n* = 8
Body weight (g)	1.45 ± 0.05	1.40 ± 0.06*	1.63 ± 0.05^††^	10.4 ± 0.5	9.49 ± 0.16*	9.74 ± 0.25	22.0 ± 0.4	21.3 ± 0.5**	21.0 ± 0.2
Brain (mg)	10.0 ± 0.5	10.1 ± 0.1	10.9 ± 0.3^†^	26.4 ± 0.5	25.5 ± 0.2	25.8 ± 0.3	26.9 ± 2.5	29.6 ± 0.4	29.3 ± 0.2
Brain %	6.88 ± 0.29	6.71 ± 0.30	6.60 ± 0.18	2.57 ± 0.11	2.68 ± 0.04	2.68 ± 0.05	1.22 ± 0.12	1.40 ± 0.02	1.40 ± 0.02

Results are expressed as mean ± SEM. Data were analyzed by one way ANOVA. *P < 0.05; **P < 0.01, compared with the SHAM offspring at the same age. ^†^*P* < 0.05; ^††^*P* < 0.01, compared to SE offspring of the same age.

#### Mitophagy Markers

**Figure 5:**
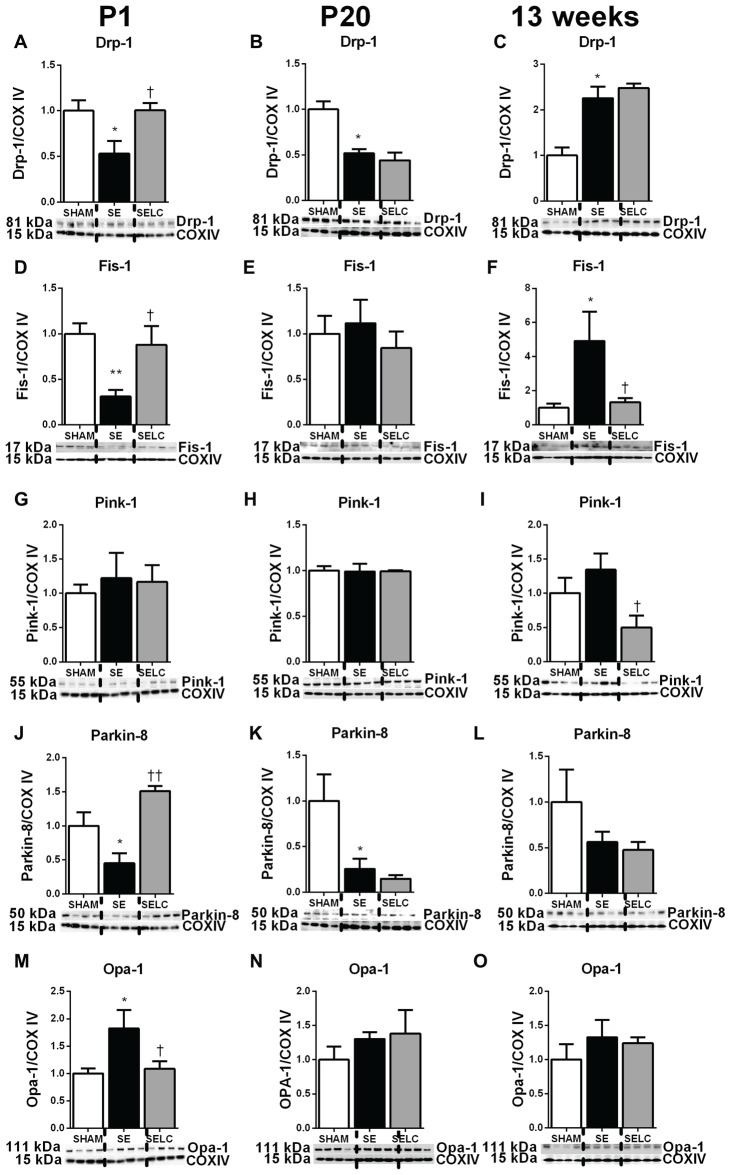
**Brain mitochondria protein levels of Drp-1 (A–C)**, Fis-1 **(D–F)**, Pink-1 **(G–I)**, Parkin **(J–L)** and Opa-1 **(M–O)** in the female SHAM, SE and SELC dams (*n* = 4) at different ages. Results are expressed as mean ± SEM. Data was analyzed by one-way ANOVA with Fisher’s LSD test. *P < 0.05, **P < 0.01, compared to SHAM; ^†^*P* < 0.05, ^††^*P* < 0.01, compared to SE. Drp-1, dynamin-related protein-1; Fis-1, fission protein-1; Pink-1, phosphatase and tensin homolog (PTEN)-induced putative kinase-1; Opa-1, optic atrophy-1; Cox IV, cytochrome c oxidase; SE, smoke exposed; SELC, SE with L-Carnitine.

At P1, mitochondrial fission markers Drp-1, Fis-1 and Parkin were significantly decreased in the SE compared to SHAM offspring (*P* < 0.05, Drp-1 and Parkin; *P* < 0.01 Fis-1; Figures 5A,D,J). Mitochondrial Opa-1 was significantly higher in the SE offspring (*P* < 0.05, Figure 5M). L-Carnitine normalized Drp-1, Fis-1 and Opal-1 levels (*P* < 0.05, Figures 5A,D,M), and tripled the level of Parkin (*P* < 0.01, Figure 5J) in the SELC compared to the SE offspring. At P20, mitochondrial Drp-1 and Parkin levels were still decreased in the SE offspring (*P* < 0.05, Figures 5B,K), which were not affected by maternal L-Carnitine treatment during gestation and lactation (Figures 5B,E,H,K,N). At 13 weeks, mitochondrial Drp-1 and Fis-1 levels were higher in the SE offspring (*P* < 0.05, Figures 5C,F), while only Fis-1 levels were normalized by maternal L-Carnitine treatment (*P* < 0.05, Figure 5F). Pink-1 protein level was significantly reduced by maternal L-Carnitine treatment, compared to SE offspring (*P* < 0.05, Figure 5I).

#### Autophagy Markers

**Figure 6:**
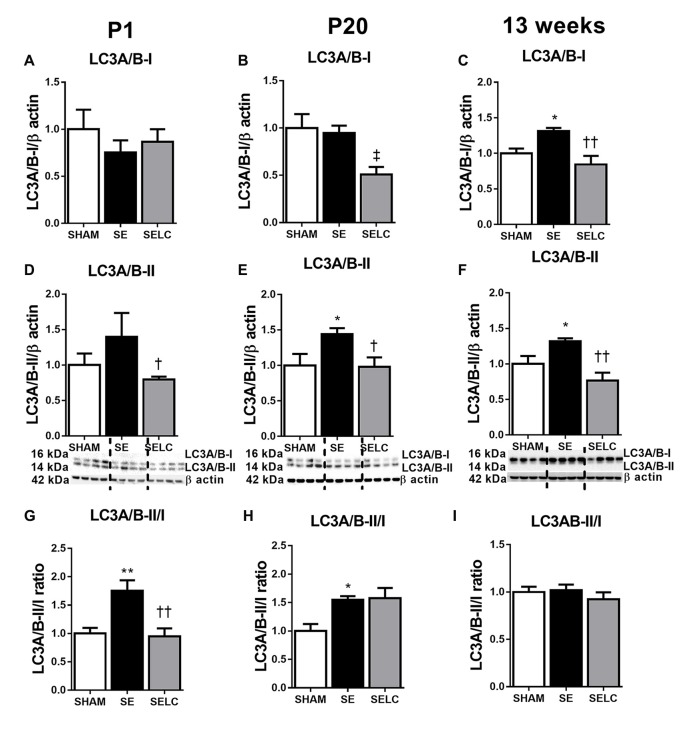
**Brain protein levels of LC3A/B-I (A–C)**, LC3A/B-II **(D–F)**, LC3A/B-II to LC3A/B-I ratio **(G–I)** in the female SHAM, SE and SELC dams (*n* = 4) at different ages. Results are expressed as mean ± SEM. Data was analyzed by one-way ANOVA with Fisher’s LSD test. *P < 0.05, **P < 0.01, compared to SHAM; ^†^*P* < 0.05, ^††^*P* < 0.01, compared to SE. ^‡^*P* < 0.05, compared to SHAM. LC3A/B, light chain 3 microtubule-associated protein A/B; SE, smoke exposed; SELC, SE with L-Carnitine.

At P1, there was a small, but not significant increase in LC3A/B-II level (Figure 6D) and significantly increased LC3A/B-II/I ratio in the SE offspring, which were both normalized by maternal L-Carnitine treatment (*P* < 0.01, Figure 6G). At P20, LC3A/B-II and LC3A/B-II/I ratios were increased in the SE offspring (*P* < 0.05, Figures 6E,H), however only LC3A/B-II level was normalized in the SELC offspring (*P* < 0.05, Figure 6E). At 13 weeks, LC3A/B-I and LC3A/B-II protein levels were increased in the SE offspring (*P* < 0.05, Figures 6C,F) and normalized by maternal L-Carnitine treatment (*P* < 0.01, Figures 6C,F).

#### Mitochondrial Functional Markers

**Figure 7:**
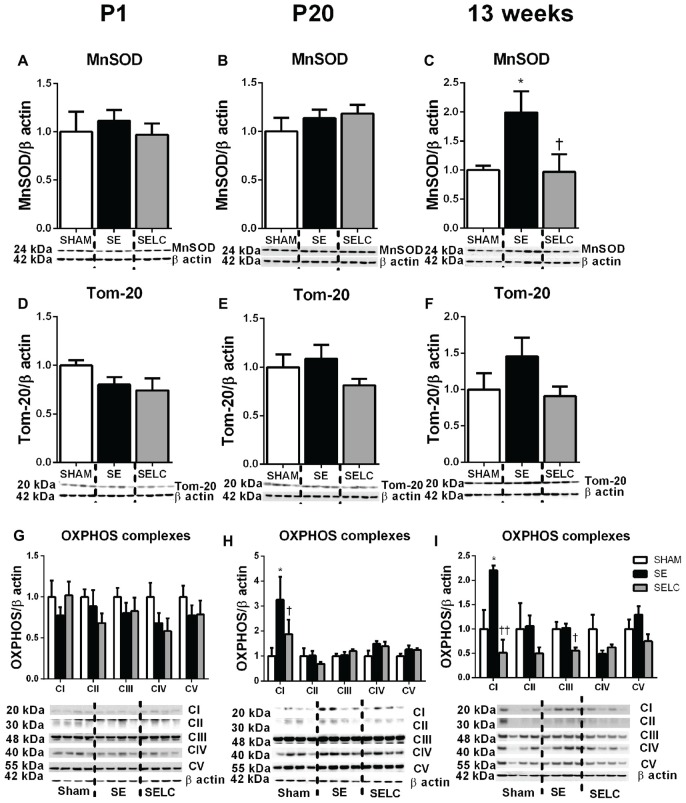
**Brain mitochondria protein levels of MnSOD (A–C)**, Tom-20 **(D–F)**, OXPHOS Complexes I-IV **(G–I)** in the female SHAM, SE and SELC dams (*n* = 4) at different ages. Results are expressed as mean ± SEM. Data was analyzed by one-way ANOVA with Fisher’s LSD test. *P < 0.05, compared to SHAM; ^†^*P* < 0.05, ^††^*P* < 0.01, compared to SE. MnSOD, manganese superoxide dismutase; Tom-20, translocase of the mitochondrial outer membrane-20; OXPHOS, oxidative phosphorylation; SE, smoke exposed; SELC, SE with L-Carnitine.

There was no significant difference in MnSOD, Tom-20 and OXPHOS complexes among the three groups at P1 (Figures 7A,D,G). At P20, only OXPHOS Complex I protein was tripled in the SE offspring, which was reduced by maternal L-Carnitine treatment (*P* < 0.05, Figure 7H) in the face of a 20% reduction in Tom-20. At 13 weeks, MnSOD level was doubled in the SE offspring, which was normalized by maternal L-Carnitine treatment (*P* < 0.05, Figure 7C). There was a 50% increase in Tom-20 in the SE offspring, although without statistical significance (Figure 7F). OXPHOS complex I level was also increased in the SE offspring (*P* < 0.05, Figure 7I); maternal L-Carnitine treatment significantly reduced OXPHOS complex I (*P* < 0.01, Figure 7I) and III (*P* < 0.05, Figure 7I) protein levels.

#### Cell Apoptosis and DNA Fragmentation

**Figure 8:**
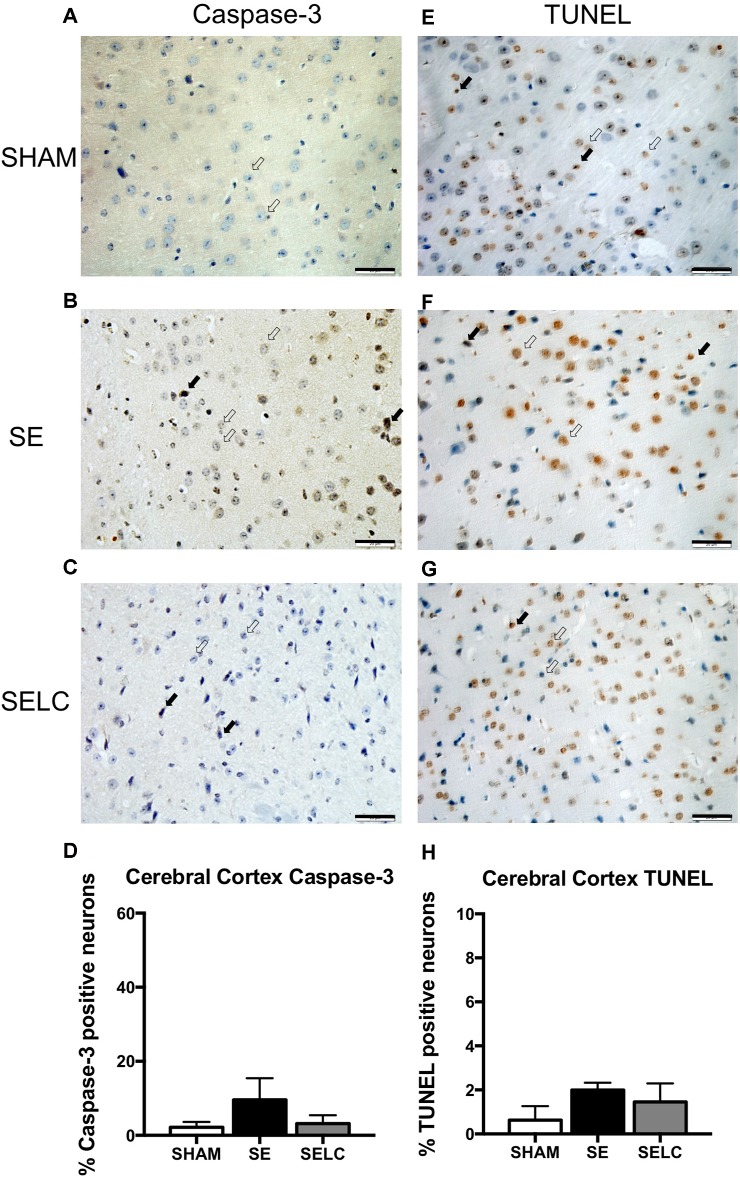
**Immunostaining for Caspase-3 staining in cerebral cortex in the female offspring at 13 weeks (*n* = 4, A–D).** Caspase-3 positive (close arrow) and caspase-3 negative (open arrow). TUNEL staining in cerebral cortex in the female offspring at 13 weeks (*n* = 4, **E–H**). TUNEL positive (close arrow) and TUNEL negative (open arrow). Scale bar = 20 μm. 40X magnification. Results are expressed as mean ± SEM. Data was analyzed by one-way ANOVA with Fisher’s LSD test. SE, smoke exposed; SELC, SE with L-Carnitine.

At 13 weeks, there were no significant changes in capase-3 and TUNEL positive cell numbers among the three female experimental groups, although cortex capase-3 and TUNEL positive cells in the SE offspring were more than doubled than the SHAM offspring (Figures 8D,H).

## Discussion

In the current study, there was a gender difference on the impacts of maternal SE on brain markers of mitophagy, the autophagosome and mitochondrial energy metabolism in offspring. Developmental changes of these markers from birth to maturity were also observed. In the male offspring, increased fission markers and reduced autophagosome markers at P1 suggesting an increase in mitochondrial damage and thereby overconsumption of the autophagosome, while data from adult offspring suggest reduced mitophagy but increased cellular damage. In the female offspring, mitochondrial fusion markers on P1 suggest increased mitochondrial regeneration, while in adults increased mitochondrial fission and autophagosome markers were observed, with high levels of MnSOD and OXPHOS complex I suggesting an increase in energy demand and oxidative stress, thereby more mitochondrial turnover (Twig et al., 2008; Lee et al., 2012). Maternal L-Carnitine supplementation during gestation and lactation partially normalized the above-mentioned changes in both male and female offspring, suggesting a possible benefit on brain health of SE offspring.

In the developing brain, substantially high energy demand increases the need for glucose, oxygen and cerebral blood flow (Hagberg et al., 2014). Mitochondria are the cellular power house and thus play important roles during brain development which is a highly energy-dependent process (Benard and Karbowski, 2009). Intrauterine environmental stress due to maternal smoking can cause adverse birth outcomes that have been well-documented (Ekblad et al., 2015). In the current study, body weight and percentage of brain weight were reduced in male SE offspring at P1 only; whereas female SE offspring remained small from birth to adulthood, without affecting percentage brain weight. This suggests that brains of the female SE offspring may be protected. This seems to be consistent with our previous observation that maternal SE enhanced risk of renal disorders in male offspring at adulthood (Al-Odat et al., 2014), but not in the female offspring (Chan et al., 2016a).

Mitophagy is a crucial process to maintain mitochondrial integrity, where damaged mitochondria can be degraded and the intact components can be recycled to generate new functional mitochondria (Benard and Karbowski, 2009). This process is achieved through fission and fusion (Westermann, 2010). Fission can fragment damaged mitochondrial parts to remove the dysfunctional components in the autophagosome by autophagy. During this process, Fis-1 is located in the outer mitochondrial membrane to recruit Drp-1 (Onoue et al., 2013), which forms a spiral to slice both the inner and outer mitochondrial membranes (Elgass et al., 2013). The damaged mitochondrial fragments are then tagged by Pink-1, followed by the recruitment of Parkin which ubiquitinates outer membrane proteins (Narendra et al., 2008). Through Pink-1 and Parkin, damaged mitochondrial fragments can be eliminated (Narendra et al., 2008) by autophagy. Autophagy activity is normally reflected by the levels of LC3A/B-I, LC3A/B-II and their ratios. LC3A/B-I is converted to LC3A/B-II (Kouno et al., 2005); which then forms autophagosomes to contain damaged organelles. LC3A/B knockout mice die shortly after birth, due to the lack of autophagy (Komatsu et al., 2005). Thus, the ratio between LC3A/B-II and LC3A/B-I or LC3A/B-II level itself can reflect autophagosome accumulation. On the other hand, the process of fusion, which is mediated by Opa-1 can facilitate healthy mitochondrial fragments to form new functional mitochondria (Kanazawa et al., 2008), which includes the exchange of mitochondrial DNA (Youle and van der Bliek, 2012). When there is increased energy demand or presence of stressor such as smoking, fusion is increased, thereby energy synthesis can be maintained (Westermann, 2012). Thus, fusion is considered as a protective mechanism during stress when energy demands are increased. Indeed, it has been found that increased mitophagy activity can improve neural survival in traumatically injured brain (Wei et al., 2015).

Here in the newborn male SE offspring, both fission and autophagosome markers were increased without changes in the fusion marker, suggesting the mitochondrial damage by maternal SE is irreparable. In the adult male SE offspring, reduced mitochondrial MnSOD suggests increased oxidative stress which may be linked to increased OXPHOS complex III, which is the major site of ROS production. This can lead to direct damage to cellular organelles including mitochondria. However, autophagosome markers are decreased which may indicate there was a defect in the removal of mitochondria fragments, while reduced mitophagy markers may indicate that there were less healthy mitochondria fragments to be recycled. Mitophagy defects have been found in neurodegenerative diseases such as Hungtington’s disease, Alzheimer’s disease and Parkinson’s disease (Schapira and Gegg, 2011; Banerjee et al., 2015), suggesting roles in maintaining neuronal integrity. Autophagy is known to block caspase-3 dependent apoptosis, a marker for cell injury (Mariño et al., 2014). As such, offspring at 13 weeks, representing adulthood, were investigated for the long term impact of maternal SE and L-carnitine supplementation. Here, markers of cell apoptosis and DNA damage were higher in adult male SE offspring, suggesting cell damage. Increase in DNA fragmentation can also increase the risk of neurodegenerative diseases (Lenardo et al., 2009). Indeed, the risk of cognitive and behavioral disorders is higher in offspring of smokers (Chen and Morris, 2007; Knopik et al., 2016; Palmer et al., 2016). Therefore, changes in brain mitophagy in the male SE offspring may predict impaired brain function later in life.

In the newborn female SE offspring, mitochondrial fission was reduced, however markers of fusion and the autophagosome were increased, which may preserve the function of mitochondria. In fact, following nutrient deprivation, healthy mitochondria elongate and fuse together (Rambold et al., 2011) to prevent degradation by autophagy (Gomes et al., 2011). A reduction in fission with increased fusion can promote the formation of elongated mitochondrial networks in order to preserve mitochondrial function (Mishra and Chan, 2014). In adulthood, both mitochondrial fission and autophagosome markers, and OXPHOS complex I were increased, suggestive of increased energy demand and mitochondrial turnover. However, the endogenous antioxidant MnSOD was increased, which may be an adaptation to counteract increased free radicals during ATP synthesis in the OXPHOS complexes. This pattern is very different from that of the male SE offspring. Opposite to what was found in male SE offspring, the apoptotic marker caspase-3 level was not changed in female’s brain suggesting better persevered cell integrity (Chandler et al., 1998). This is consistent with unchanged brain inflammatory and oxidative stress markers in our previous study using the same model (Chan et al., 2016a). The marginal change in TUNEL staining somewhat mirrors the changes in mitophagy markers in the female’s brain, suggesting the mitophagy regulation may play a critical role in cell integrity.

There is a significant gender difference in response to maternal SE as shown by us previously (Chan et al., 2016a,b), where female offspring seem to be more protected from increased brain inflammatory and oxidative stress markers by maternal SE. Here in addition to mitophagy and autophagy markers, cell injury seems to be more pronounced in the male offspring. One potential mechanism is the sex hormone estrogen. Estrogen has been shown to bear neuroprotective property (Brann et al., 2007). During cerebral ischemia, the blockage of estrogen receptors can increase infarction size following carotid artery occlusion in female rats (Sawada et al., 2000); whereas the increase in estrogen was shown to increase the expression of the anti-apoptotic gene (B-cell lymphoma 2) in mice with cerebral ischemia (Dubal et al., 1999). Thus it is explainable why the cell injury markers were less changed in female SE offspring.

Complete smoking cessation is difficult to achieve during pregnancy, especially in certain communities where maternal smoking rates are particularly high (Chertok and Archer, 2015; Glover et al., 2016). A pregnancy supplement is more feasible approach, in comparison to difficulties introducing behavioral changes (i.e., smoking cessation) to improve fetal health outcomes in this population. The antioxidant L-Carnitine, is approved by the US Food and Drug Administration to treat Carnitine deficiency in dialysis patients (Guarnieri et al., 2001). It is also an over-the-counter supplement for athletes and bodybuilders and also shown to help weight loss in adults (Pooyandjoo et al., 2016). We have previously shown that L-Carnitine supplementation during gestation and lactation can reverse the detrimental impact of maternal SE on renal development and function in offspring (Nguyen et al., 2015). In the current study, SE offspring displayed smaller birth weight and body weight at adulthood, which is consistent with our previous observations (Chan et al., 2016a,b). Maternal L-Carnitine supplementation normalized body weight at birth and thereafter that at adulthood in the SE offspring of both genders, as well as small brain weight at birth in the males. This suggests that the protection in the offspring is most likely due to the maternal intrauterine effect on fetal growth, which further resulted in normal postnatal growth. However, the exact mechanism requires further investigation. Interestingly, the changes in mitochondrial fission and fusion makers, as well as OXPHOS complex and MnSOD were partially normalized in the SE offspring of both genders, as well as cell injury markers Caspase-3 and TUNEL. There is consistent with the observations in kidney development and function in the same cohort of SE offspring (Nguyen et al., 2015). L-Carnitine has been shown to regulate both mitochondria and autophagy processes (Hagen et al., 1998; Long et al., 2009; Shenk et al., 2009; Marcovina et al., 2013; Zhu et al., 2015). However, it is difficult to separate these two aspects in the current study design. Thus, maternal L-Carnitine supplementation during gestation and lactation may improve the health outcomes in SE offspring in the long term.

In conclusion, maternal SE impaired brain mitochondrial markers of fission and fusion and increased oxidative stress in both genders, with the effect delayed in the females. Our study confirms the benefits of L-Carnitine use in high-risk pregnancies to improve potential health outcomes in offspring by replenishing mitophagy function in brains of offspring.

## Author Contributions

HC, NMJ and SS designed the study. YLC, HC, and IA-O performed all the experiments. YLC, BGO, SS, NMJ and HC contributed to the writing of the main manuscript text, and YLC prepared Figures 1–8 and Tables 1, 2. All authors reviewed the manuscript.

## Conflict of Interest Statement

The authors declare that the research was conducted in the absence of any commercial or financial relationships that could be construed as a potential conflict of interest.

## Abbreviations

Cox: cytochrome c oxidase subunit
Drp-1: dynamin-related protein-1
Fis-1: fission protein
LC3A/B: light chain 3 microtubule associated protein A/B
MnSOD: manganese superoxide dismutase
Opa-1: optic atrophy 1
OXPHOS: oxidative phosphorylation
P: postnatal
Pink-1: phosphatase and tensin homolog induced putative kinase
ROS: reactive oxygen species
SE: cigarette smoke exposed
SELC: SE breeders supplied with L-Carnitine
Tom-20: translocase of outer membrane-20.
